# Supplementary material for: Biosynthetic production of anticoagulant heparin polysaccharides through metabolic and sulfotransferases engineering strategies
Source: Nat Commun. 2024 May 4;15:3755. doi: 10.1038/s41467-024-48193-5 (PMC11069525; doi:10.1038/s41467-024-48193-5)
Supplement: Supplementary file 1 — Supplementary Information [file 41467_2024_48193_MOESM1_ESM.pdf]

Biosynthetic production of anticoagulant heparin polysaccharides through metabolic and sulfotransferases engineering strategies

Deng *et al.*

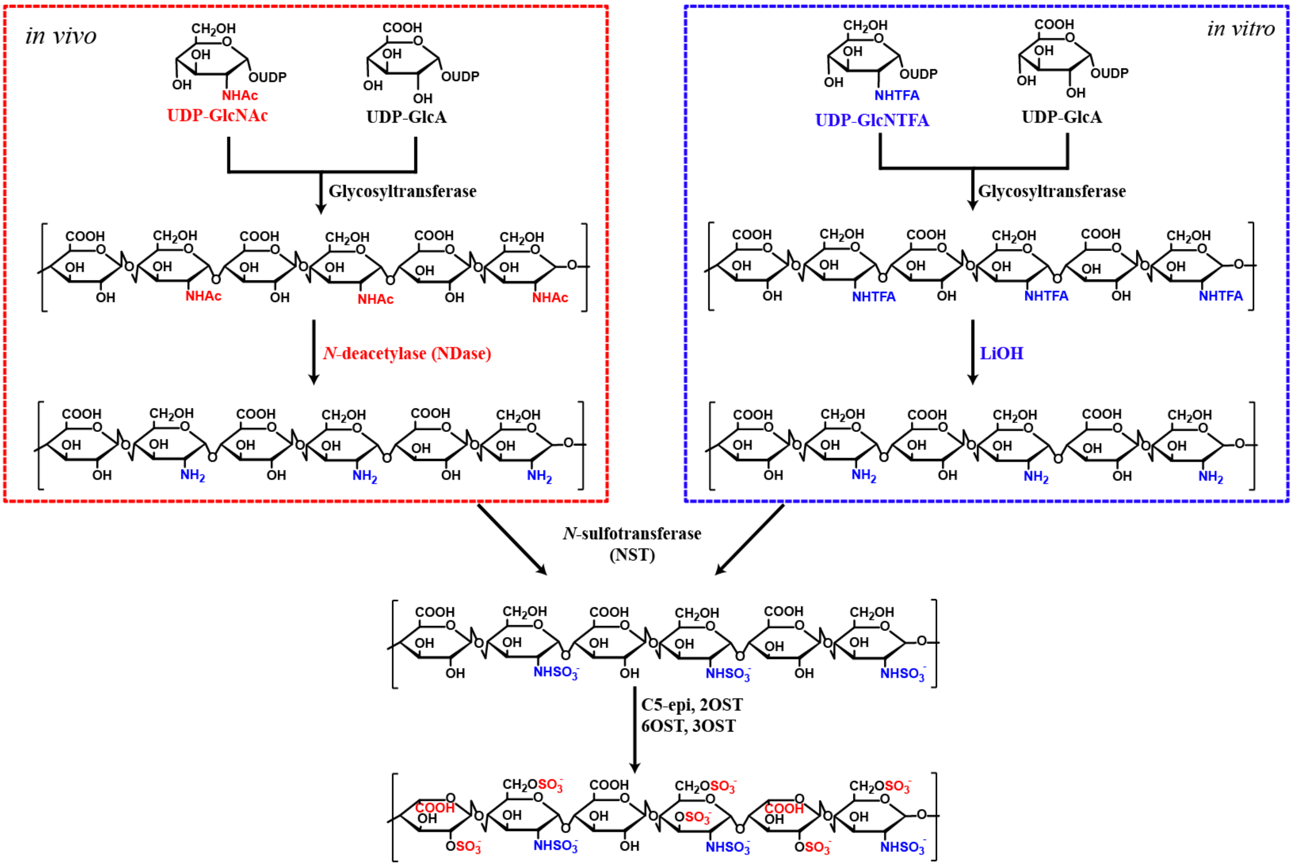

Supplementary Figure 1. Schematic representation of the difference in *N*-deacetylation at the *N*-sites between the *in vivo* heparin biosynthesis pathway and the *in vitro* heparin chemoenzymatic synthesis strategy.

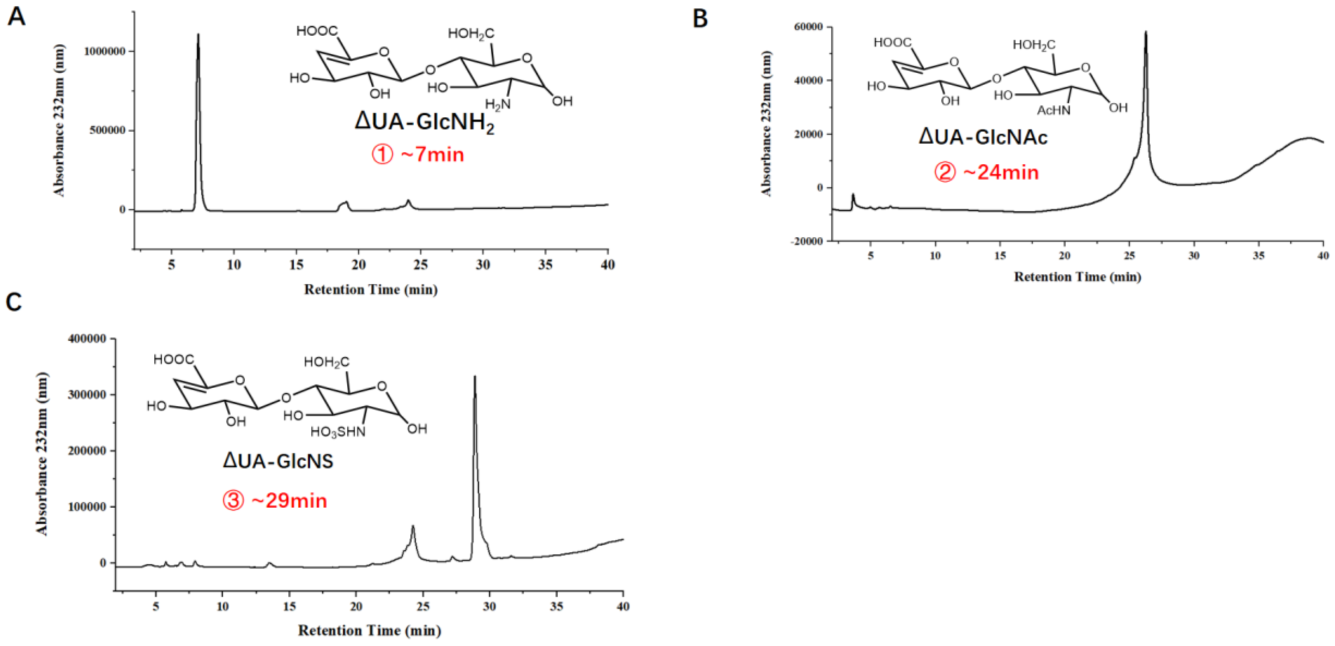

Supplementary Figure 2. PAMN-HPLC analysis of disaccharide components of bioengineering heparosan purified by DEAE chromatography. (A) ΔUA-$GlcNH_2$. (B) ΔUA-GlcNAc. (C)ΔUA-GlcNS. Source data are provided as a Source Data file.

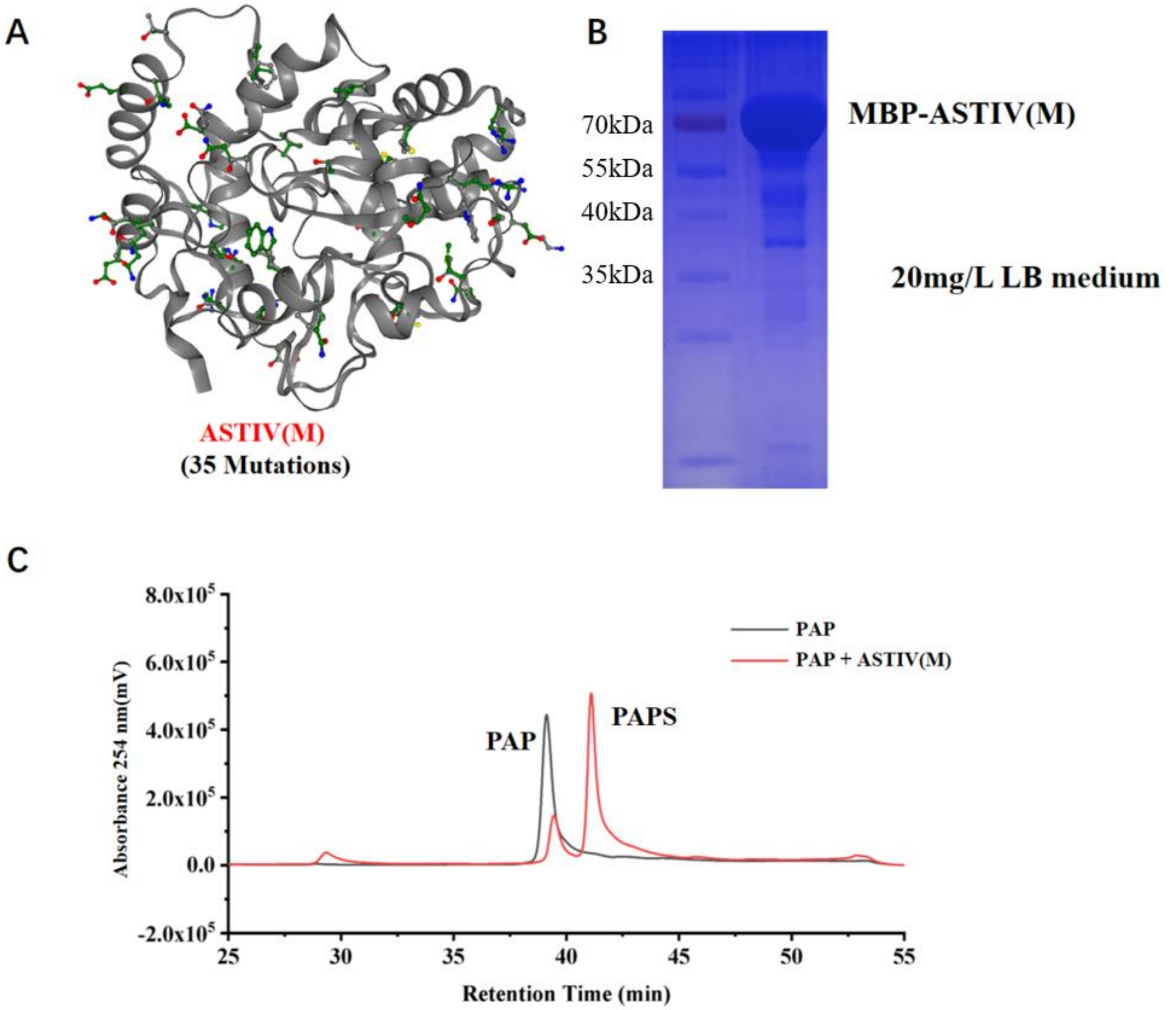

Supplementary Figure 3. Activity analysis of ASTIV(M). (A) The positions of 35 mutations in the crystal structure of ASTIV(M). The protein structure of ASTIV was obtained through homology modeling using human SULT1A1 (PDB: 3U3R) as the template, and SWISS-MODEL software. (B) SDS-PAGE analysis of purified ASTIV(M). Approximately 20 mg of MBP-ASTIV(M) protein could be purified per liter of LB medium. The protein molecular weight markers were Thermo Scientific Prestained Protein Molecular Weight Standard, 10 to 180 kDa. The protein band corresponds to the expected molecular weight: MBP–ASTIV(M), 77.5 kDa. (C) Determination of enzyme activity of ASTIV(M). Source data are provided as a Source Data file.

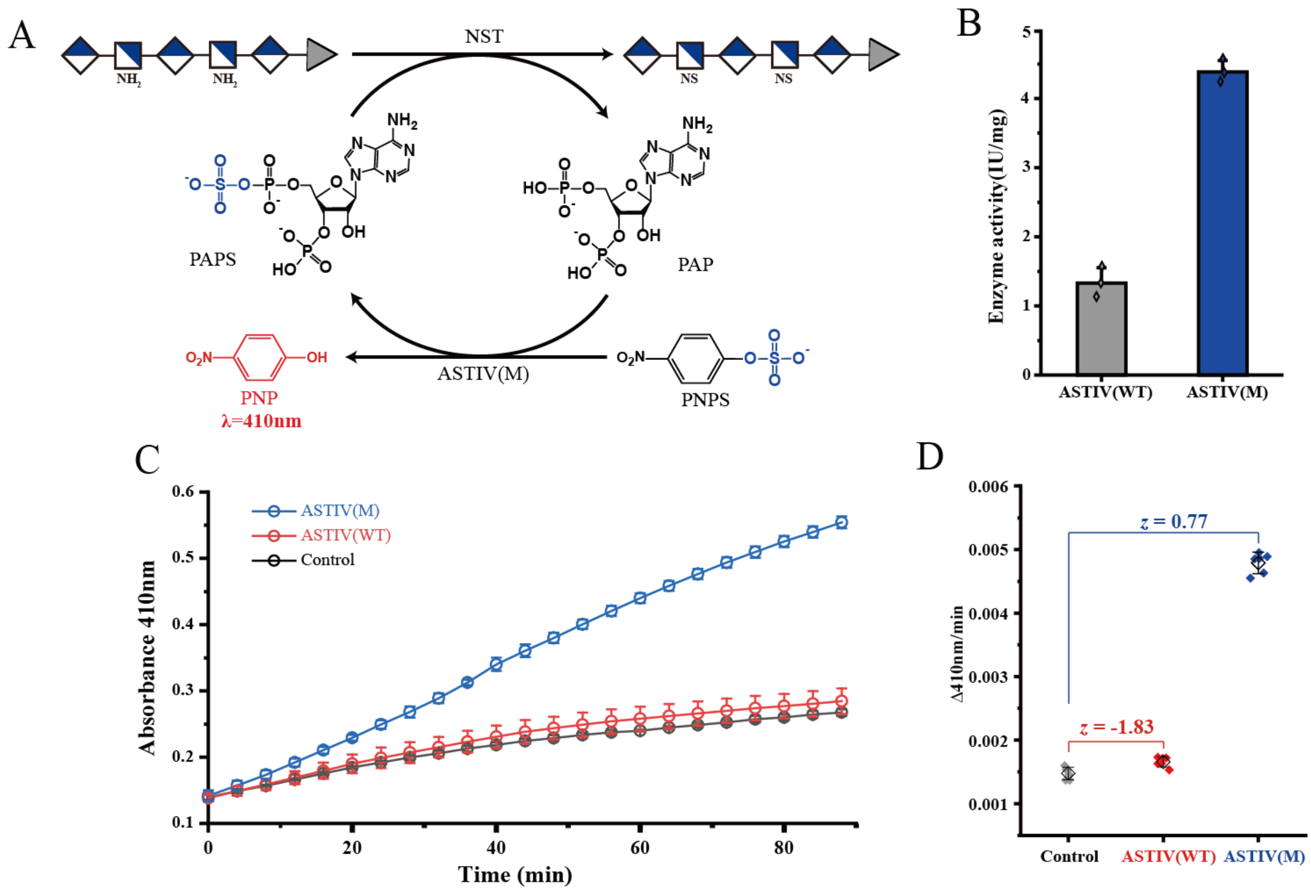

Supplementary Figure 4. Application of ASTIV(M) in high-throughput screening. (A) Sulfotransferase screening system based on ASTIV(M). (B) Comparison of activity between ASTIV(M) and wild-type ASTIV. Conduct the experiment using three independent samples. (C) Kinetic determination of ASTIV(M) and wild-type ASTIV at 410 nm in the screening system. (D) Comparison of the z-factor between ASTIV(M) and wild-type ASTIV. The z-factor of ASTIV(M) reached 0.77, which is >0.5, meeting the requirements for high-throughput screening. Source data are provided as a Source Data file.

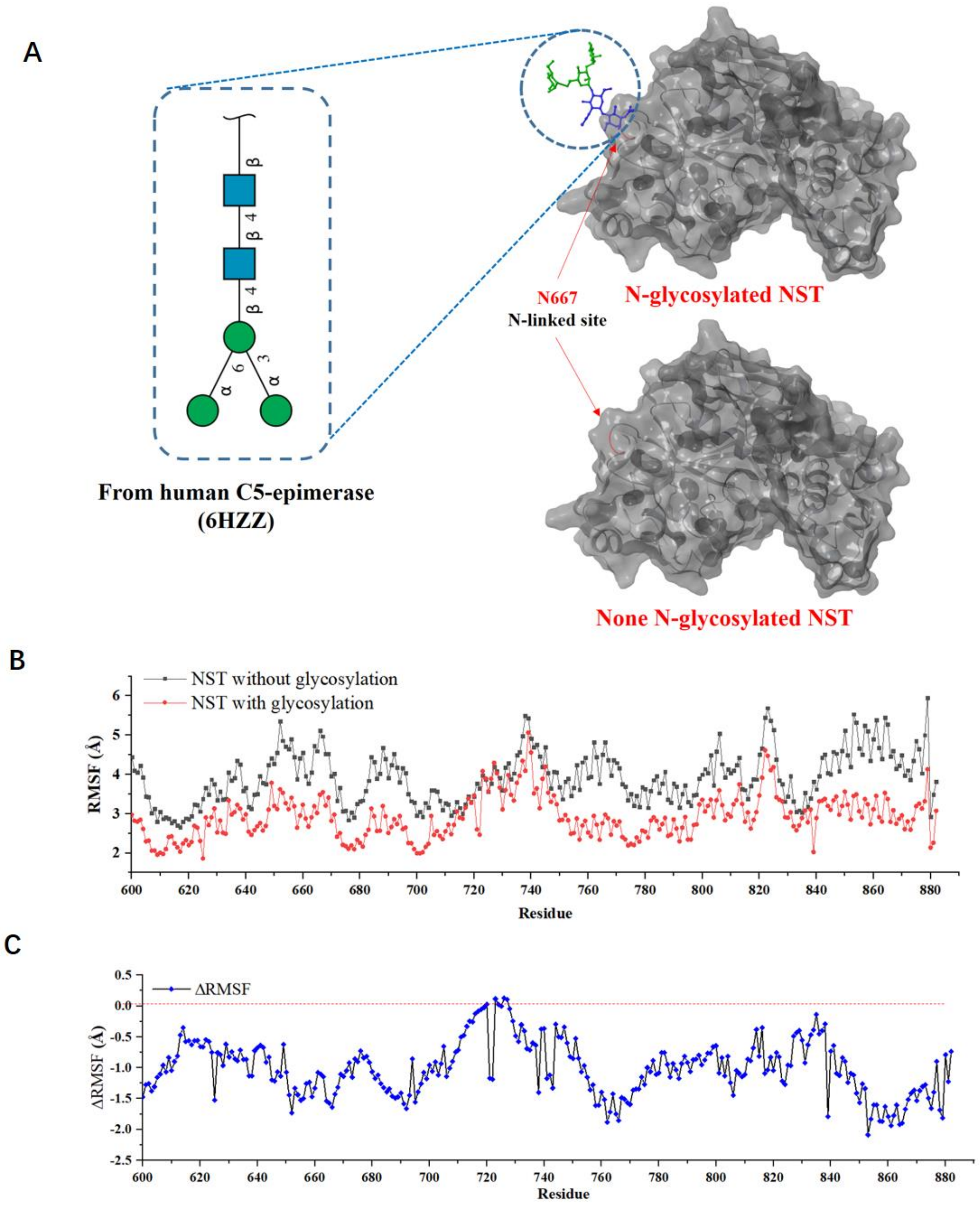

Supplementary Figure 5. MD simulations of NST protein structure with or without glycosylation. (A) Grafting of the pentasaccharide $GlcNAc_2Man_3$ from the human C5-epimerase structure onto the *N*-glycosylation site N667 of NST. (B) RMSF results for NST with and without glycosylation after 20 ns of NPT simulation using Amber. (C) Difference in RMSF of NST protein with and without glycosylation. Source data are provided as a Source Data file.

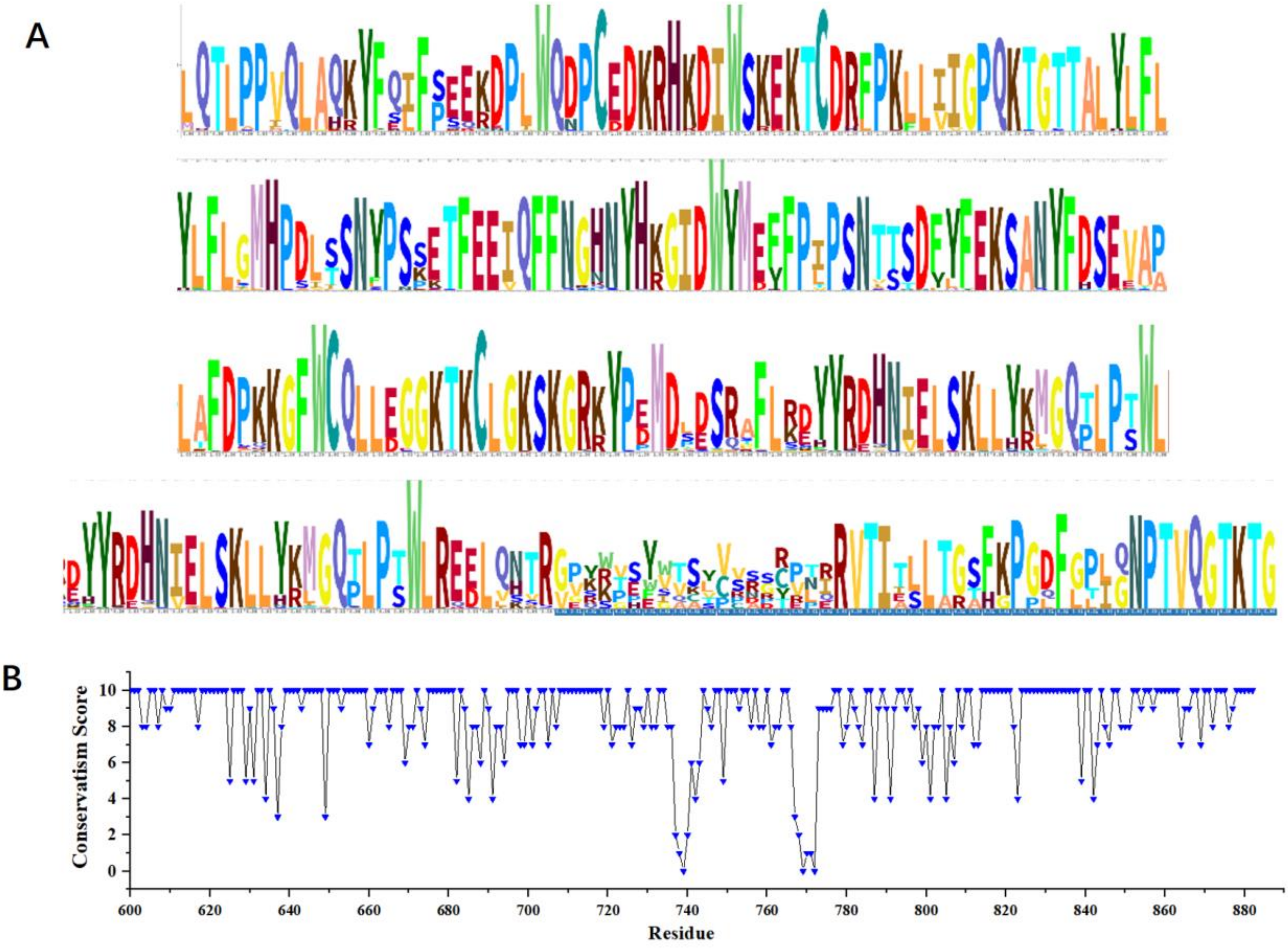

Supplementary Figure 6. Multiple sequence alignment of *N*-sulfotransferases and homologous sequences. (A) Weblogo seqlogo of *N*-sulfotransferases and homologous sequences from BLAST. The clustalw program was used to compare the multiple sequences (https://www.genome.jp/tools-bin/clustalw), and then the graph visualization used skylign (http://skylign.org/). (B) Conservation analysis of *N*-sulfotransferases and homologous sequences. The evaluation values range from 0 to 10, with 10 indicating complete amino acid conservation at that position. Source data are provided as a Source Data file.

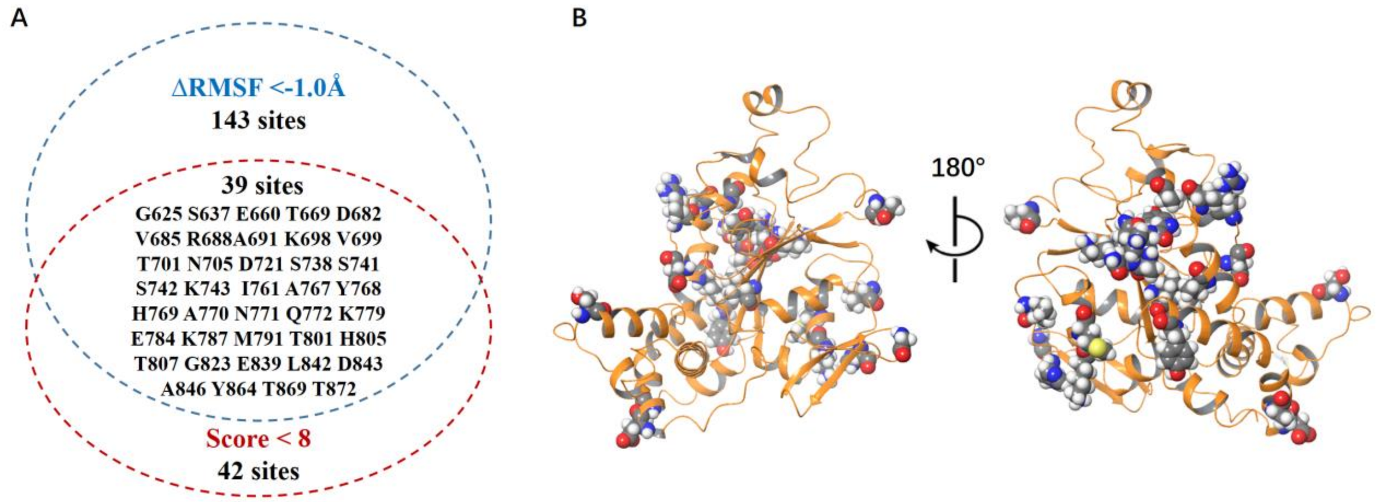

Supplementary Figure 7. Illustration of amino acid residues in the mutation range for NST. (A) A total of 143 residues met the criteria of ΔRMSF <1.0, as determined by MD simulation (Supplementary Figure 5). A total of 42 residues met the criteria of conservation analysis score <8 (Supplementary Figure 6). The intersection of the two sets resulted in 39 identified residues, as shown in this figure. Mutations in NST were performed at these 39 positions. (B) The positions of the 39 mutation sites in the NST crystal structure.

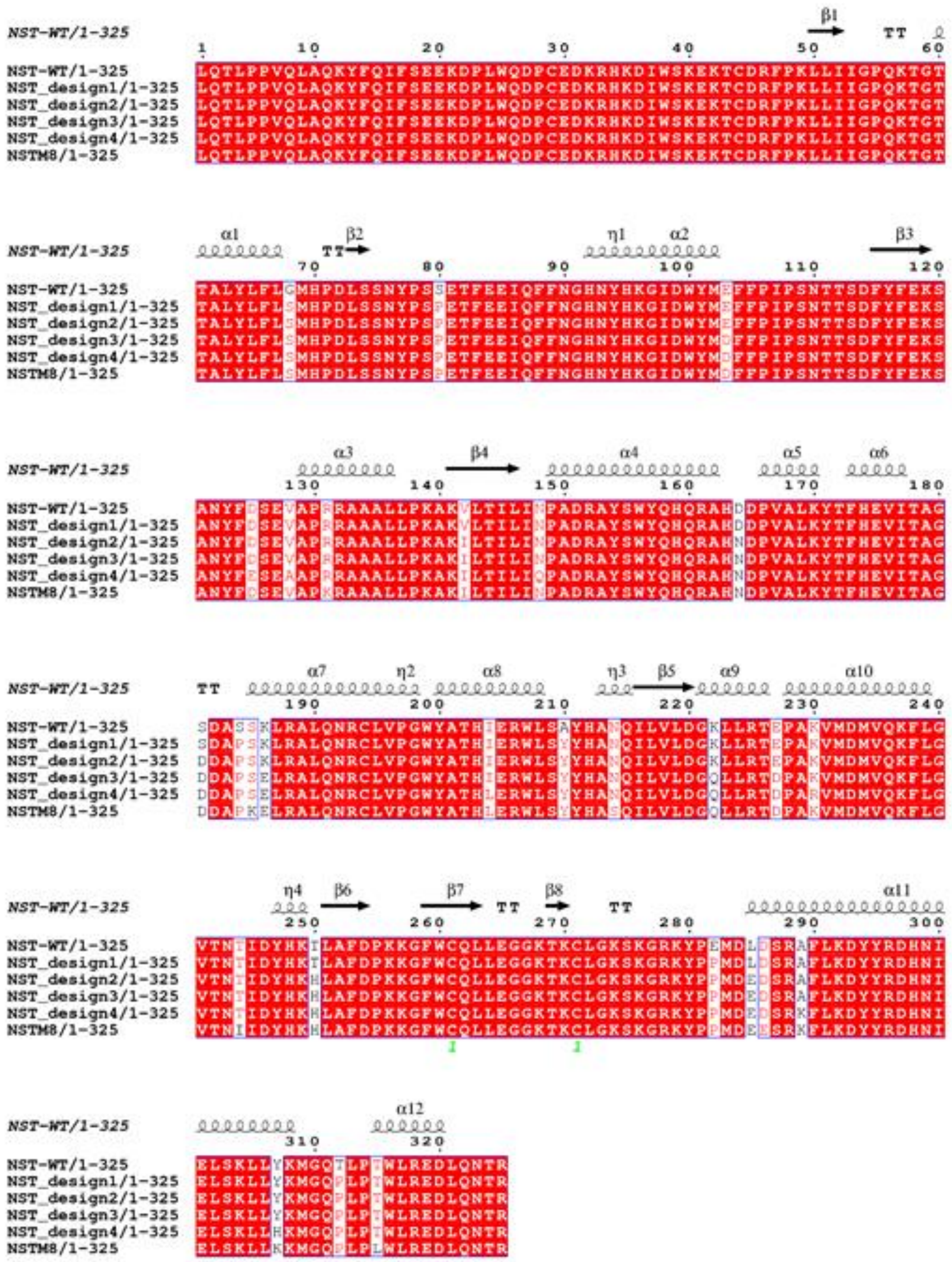

Supplementary Figure 8. Sequence alignment of wild-type NST and mutants. Strictly conserved residues are highlighted by a red background, and conservatively-substituted residues are boxed. The secondary structure of NST is shown above the aligned sequences.

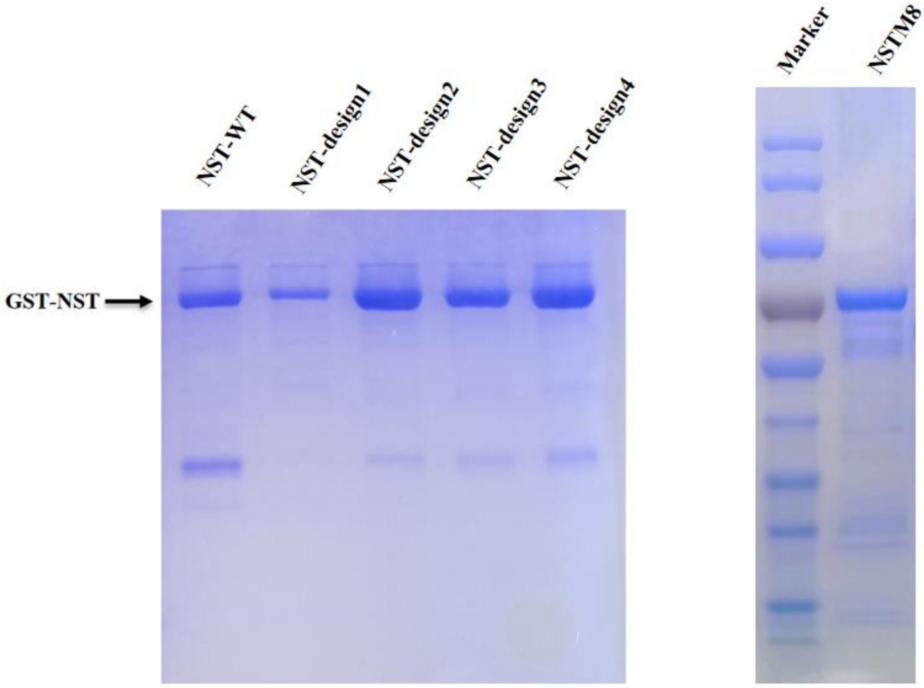

Supplementary Figure 9. SDS-PAGE analysis of purified proteins. The protein molecular weight markers were Thermo Scientific Prestained Protein Molecular Weight Standard, 10 to 180 kDa. Each band corresponds to the expected molecular weight: glutathione-*S*-tagged–NST and its mutants, 69.62 kDa. Source data are provided as a Source Data file.

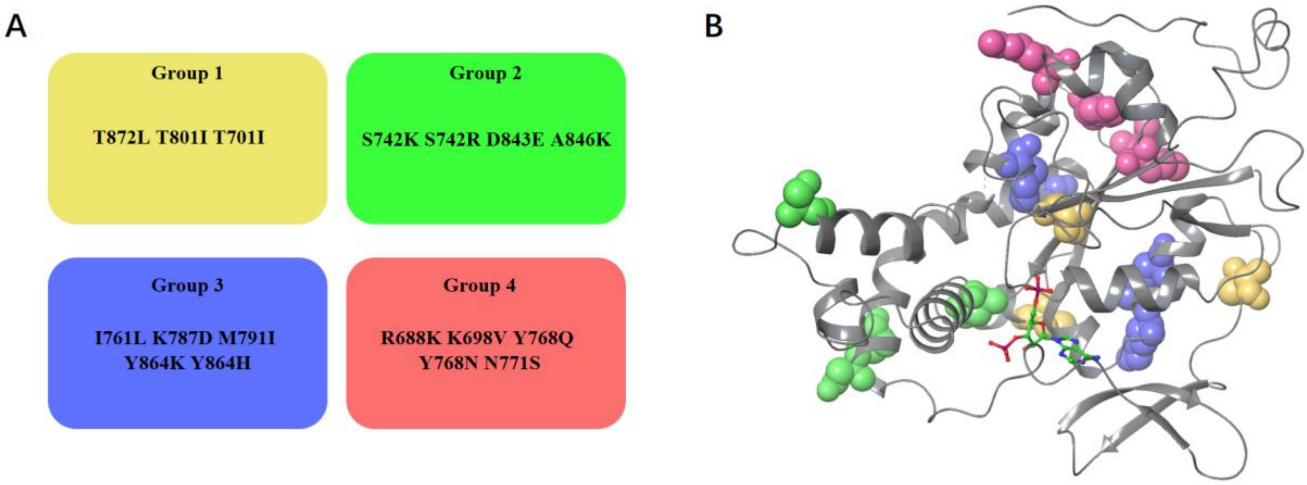

Supplementary Figure 10. Amino acid grouping during the second round of the iterative evolution process. (A) A series of single point mutation stability-enhancing sites designed on the basis of NSTM1 was divided into four groups based on spatial positional relationships. Yellow, group 1, T872L, T801I, and T701I; green, group 2, S742K, S742R, D843E, and A846K; blue, group 3, I761L, K787D, M791I, Y864K, and Y864I; red, group 4, R688K, K698V, Y768Q, Y768N, and N771S.(B) Diagram of Amino Acid Grouping in NST protein structure. Yellow, group 1; green, group 2; blue, group 3; red, group 4.

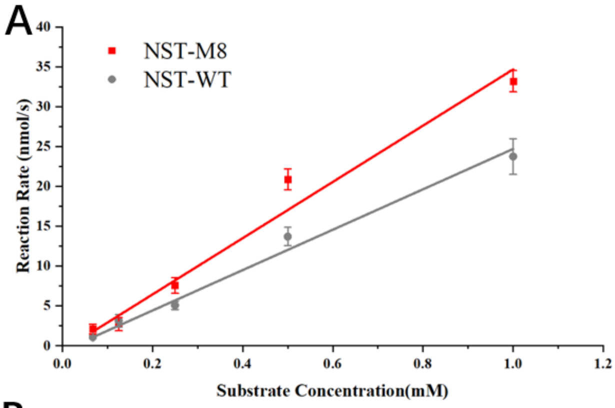

Enzyme	V_{max} (nmol/s)	K_m (μM)	k_{cat} (/s)	k_{cat}/K_m (μM/s)
NST-WT	15.87	100.5	378	3.76
NST-M8	22.22	133.4	740	5.55

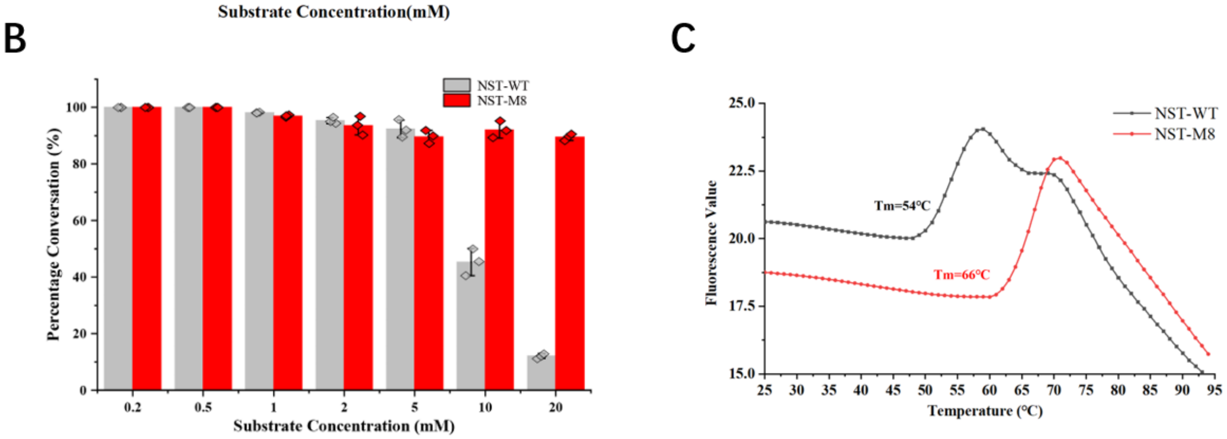

Supplementary Figure 11. The mutant NST-M8 exhibits high stability. (A) Enzymatic reaction kinetics of wild-type NST(NST-WT) and NST-M8. (B) The maximum tolerable substrate concentration of wild-type NST and NST-M8. (C) The melting temperature of wild-type NST and NST-M8. Source data are provided as a Source Data file.

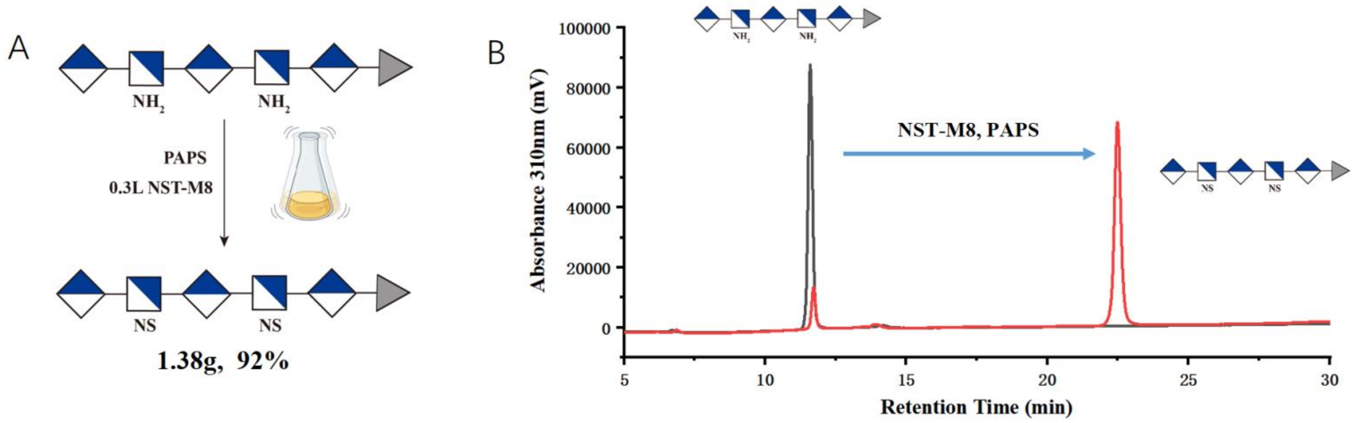

Supplementary Figure 12. Synthesis of *N*-site-modified oligosaccharides on a gram-scale. (A) Schematic diagram of the synthesis of *N*-site modified pentasaccharides. (B) *N*-sulfated pentasaccharide synthesis determined by PAMN-HPLC analysis. Source data are provided as a Source Data file.

A

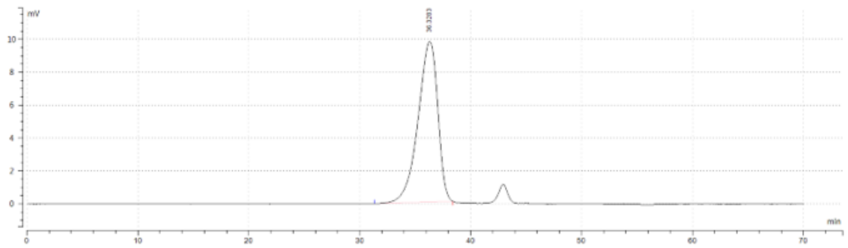

RT(min)	lgMp	lgMw	lgMn	Mp	Mw	Mn
36.328	4.3	4.3	4.3	11205	10127	9669

B

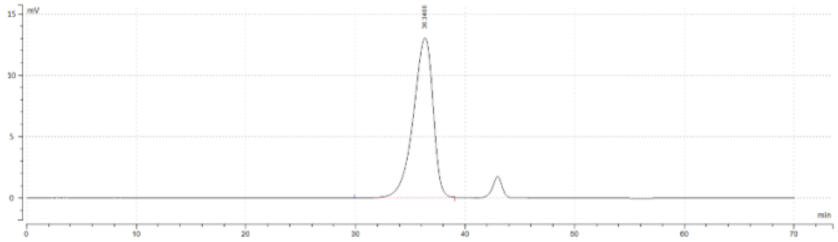

RT(min)	lgMp	lgMw	lgMn	Mp	Mw	Mn
36.347	4.3	4.3	4.3	11014	9945	9491

C

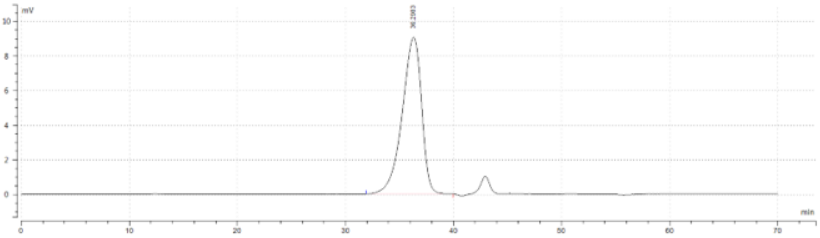

RT(min)	lgMp	lgMw	lgMn	Mp	Mw	Mn
36.298	4.3	4.3	4.3	11509	10418	9953

D

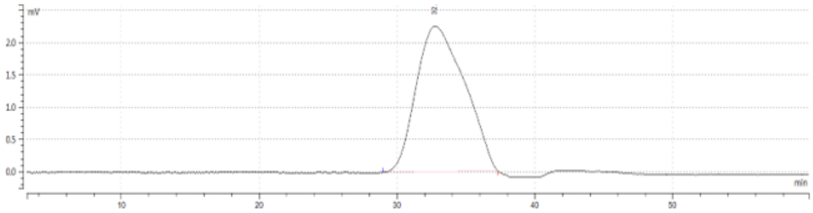

RT (min)	lgMp	lgMw	lgMn	Mp	Mw	Mn
32.747	4.2	4.2	4.2	16477	15835	15377

Supplementary Figure 13. The molecular weight of bioengineered heparin determined by high-performance gel permeation chromatography. (A) Molecular weight determination of engineered K5 polysaccharides from cells cultured using 100 μg/mL $Ac_4GlcNTFA$. (B) Molecular weight determination of engineered K5 polysaccharides from cells cultured using 200 μg/mL$Ac_4GlcNTFA$. (C) Molecular weight determination of engineered K5 polysaccharides from cells cultured using 500 μg/mL $Ac_4GlcNTFA$. (D) Molecular weight determination of K5EH. Note: M_n represents the number average molecular weight, M_p represents the peak position molecular weight, M_w represents the weight average molecular weight, and the unit of molecular weight is Dalton (Da). Source data are provided as a Source Data file.

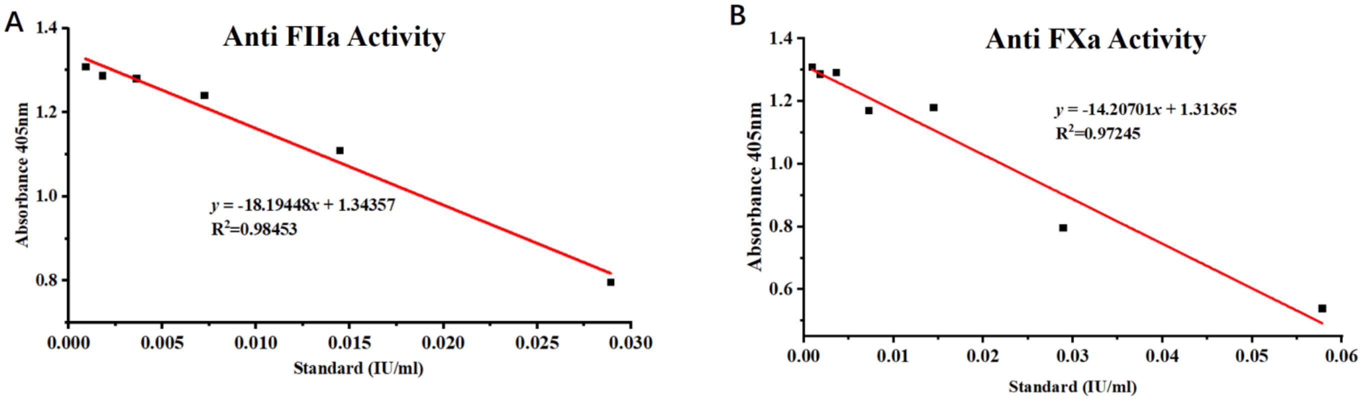

Supplementary Figure 14. The standard curve of anti-FIIa and anti-FXa activity of bioengineered heparin. (A) Standard curve for anti-FIIa activity. (B) Standard curve for anti-FXa activity. Source data are provided as a Source Data file.

Supplementary Table 1. Strains used in this study.

Strain	Feature	Source
E. coli K5ASSH	*Escherichia coli* O10:K5:H4; KfiA and glmS deleted, carrying pACYDuet-POXB20-AGX1-NahK	[1]
NST(and mutations)	Origami B(DE3) carrying pGEX-4T-1-NST	This study
C5-epi	pGro7/Origami B(DE3) carrying pMAL-c5x-C5-epi	[2]
2OST	Origami B(DE3) carrying pMAL-c5x-2OST	[3]
6OST-1	Origami B(DE3) carrying pMAL-c5x-6OST-1	[4]
3OST-1	Origami B(DE3) carrying pMAL-c5x-3OST-1	[5]
ASTIV and ASTIV(M)	Origami B(DE3) carrying pMAL-c5x- ASTIV	This study
HepI, HepII, and HepIII	BL21(DE3) carrying pET21b (+)-Hep	[6]
z-NSTWT and z-NSTM8	Origami B(DE3) carrying pGEX-4T-1-NST with a Zbasic2 tag at the *N*-terminus	This study

Supplementary Table 2. Sequences of key enzymes.

Protein	Sequence
NST(wild type)	LQTLPPVQLAQKYFQIFSEEKDPLWQDPCEDKRHKDIWSKEKTCDRFPKLLIIGPQKTGTTALYLFLG<br>MHPDLSSNYPSSETFEEIQFFNGHNYHKGIDWYMEFFPIPSNTTSDFYFEKSANYFDSEVAPRRAAAL<br>LPKAKVLTILINPADRAYSWYQHQRAHDDPVALKYTFHEVITAGSDASSKLRALQNRCLVPGWYAT<br>HIERWLSAYHANQILVLDGKLLRTEPAKVMDMVQKFLGVTNTIDYHKTLAFDPKKGFWCQLLEGG<br>KTKCLGKSKGRKYPEMDLDSRAFLKDYYRDHNIELSKLLYKMGQTLPTWLREDLQNTR*
NST_design1 (6 mutations)	LQTLPPVQLAQKYFQIFSEEKDPLWQDPCEDKRHKDIWSKEKTCDRFPKLLIIGPQKTGTTALYLFLS<br>MHPDLSSNYPSPETFEEIQFFNGHNYHKGIDWYMEFFPIPSNTTSDFYFEKSANYFDSEVAPRRAAAL<br>LPKAKVLTILINPADRAYSWYQHQRAHDDPVALKYTFHEVITAGSDAPSKLRALQNRCLVPGWYAT<br>HIERWLSYYHANQILVLDGKLLRTEPAKVMDMVQKFLGVTNTIDYHKTLAFDPKKGFWCQLLEGG<br>KTKCLGKSKGRKYPPMDLDSRAFLKDYYRDHNIELSKLLYKMGQPLPTWLREDLQNTR*
NST_design2 (11 mutations)	LQTLPPVQLAQKYFQIFSEEKDPLWQDPCEDKRHKDIWSKEKTCDRFPKLLIIGPQKTGTTALYLFLS<br>MHPDLSSNYPSPETFEEIQFFNGHNYHKGIDWYMEFFPIPSNTTSDFYFEKSANYFDSEVAPRRAAAL<br>LPKAKILTILINPADRAYSWYQHQRAHNDPVALKYTFHEVITAGDDAPSKLRALQNRCLVPGWYAT<br>HIERWLSYYHANQILVLDGKLLRTEPAKVMDMVQKFLGVTNTIDYHKHLAFDPKKGFWCQLLEGG<br>KTKCLGKSKGRKYPPMDEDSRAFLKDYYRDHNIELSKLLYKMGQPLPTWLREDLQNTR*
NST_design3 (15 mutations)	LQTLPPVQLAQKYFQIFSEEKDPLWQDPCEDKRHKDIWSKEKTCDRFPKLLIIGPQKTGTTALYLFLS<br>MHPDLSSNYPSPETFEEIQFFNGHNYHKGIDWYMDFFPIPSNTTSDFYFEKSANYFDSEVAPRRAAAL<br>LPKAKILTILINPADRAYSWYQHQRAHNDPVALKYTFHEVITAGDDAPSELRALQNRCLVPGWYATH<br>IERWLSYYHANQILVLDGQLLRTDPAKVMDMVQKFLGVTNTIDYHKHLAFDPKKGFWCQLLEGGK<br>TKCLGKSKGRKYPPMDEDSRAFLKDYYRDHNIELSKLLYKMGQPLPTWLREDLQNTR*
NST_design4 (22 mutations)	LQTLPPVQLAQKYFQIFSEEKDPLWQDPCEDKRHKDIWSKEKTCDRFPKLLIIGPQKTGTTALYLFLS<br>MHPDLSSNYPSPETFEEIQFFNGHNYHKGIDWYMDFFPIPSNTTSDFYFEKSANYFESEAAPRRAAAL<br>LPKAKILTILIQPADRAYSWYQHQRAHNDPVALKYTFHEVITAGDDAPSELRALQNRCLVPGWYATH<br>LERWLSYYHANQILVLDGQLLRTDPARVMDMVQKFLGVTNTIDYHKHLAFDPKKGFWCQLLEGGK<br>TKCLGKSKGRKYPPMDEDSRKFLKDYYRDHNIELSKLLHKMGQPLPTWLREDLQNTR*
NSTM8 (24 mutations)	LQTLPPVQLAQKYFQIFSEEKDPLWQDPCEDKRHKDIWSKEKTCDRFPKLLIIGPQKTGTTALYLFLS<br>MHPDLSSNYPSPETFEEIQFFNGHNYHKGIDWYMDFFPIPSNTTSDFYFEKSANYFDSEVAPKRAAAL<br>LPKAKILTILINPADRAYSWYQHQRAHNDPVALKYTFHEVITAGDDAPKELRALQNRCLVPGWYAT<br>HLERWLSYYHASQILVLDGQLLRTDPAKVMDMVQKFLGVTNIIDYHKHLAFDPKKGFWCQLLEGG<br>KTKCLGKSKGRKYPPMDEESRKFLKDYYRDHNIELSKLLKKMGQPLPLWLREDLQNTR*
ASTIV	MEFSRPPLVHVKGIPLIKYFAETIGPLQNFTAWPDDLLISTYPKSGTTWMSEILDMIYQGGKLEKCGR<br>APIYARVPFLEFKCPGVPSGLETLEETPAPRLLKTHLPLSLLPQSLLDQKVKVIYIARNAKDVVVSYYN<br>FYNMAKLHPDPGTWDSFLENFMDGEVSYGSWYQHVKEWWELRHTHPVLYLFYEDIKENPKREIKKI<br>LEFLGRSLPEETVDSIVHHTSFKKMKENCMTNYTTIPTEIMDHNVSPFMRKGTTGDWKNTFTVAQNE<br>RFDAHYAKTMTDCDFKFRCEL*
ASTIV(M) (35 mutations)	MEFSRPPLVHVKGIPLIKYFAETWGQLQNFQARPDDLLIATYPKSGTTWLSEILDMIYQGGDLEKCQR<br>APIYNRVPFLEFKCPGVPSGLEQLEEMPSPRLLKTHLPLQLLPQSLLDQKCKVIYIARNAKDVVVSYY<br>HFYRMNKLHPDPGTWDEFLEKFMDGKVAYGSWYDHVKEWWELRHTHPILYLFYEDMKENPKREI<br>KKILEFLGKSLPEETVDKIVHHTSFDKMKENPMTNYTTIPTEIMDHSVSPFMRKGTVGDWKNHFTVA<br>QNERFDEHYAKKMADCDLKFRCEL*

Supplementary Table 3. Virtual mutation results calculated by Rosetta_ddg based on NST-M1.

	A	C	D	E	F	G	H	I	K	L	M	N	P	Q	R	S	T	V	W	Y
T669	0.2	0.06	0.07	0.03	0.17	0.18	-0.05	-0.05	0.16	-0.03	0.01	0.12	0.01	0.08	0.09	0.17	0	-0.06	0.32	0.18
D682	0.34	0.31	0	0.33	0.17	0.93	-0.08	-0.3	0.38	0.42	0.27	0.06	0.89	0.09	0.16	0.54	-0.09	0.37	0.56	0.45
V685	1.43	1.3	0.82	0.44	0.64	2.22	0.43	0.65	0.56	0.77	0.77	0.92	1.52	0.61	0.77	1.16	0.65	0	0.46	0.54
R688	-0.13	0.55	0.33	0.16	0.61	0.94	0.33	0.11	-0.74	0.12	0.35	0.34	0.7	-0.07	0	0.35	0.28	0.23	0.55	0.24
A691	0	0.64	0.86	0.72	0.64	0.87	0.76	0.76	0.44	0.67	0.4	0.77	1.75	0.33	0.27	0.57	0.68	0.71	0.2	0.48
K698	-0.46	0.23	0.83	0.95	0.82	1.38	0	-0.19	0	0.85	0.71	0.48	1.23	0.38	0.92	0.75	-0.26	-0.19	0.52	0.67
T701	1.6	0.18	3	3.29	-0.28	2.56	1.2	-0.77	2.84	-0.23	0.36	2.32	2.72	2.29	1.89	2.05	0	-0.74	0.14	-0.06
N705	-0.77	1.07	-0.68	2.07	1.24	-0.21	0.63	1.24	2.05	0.84	1.05	0	0.68	1.79	1.55	0.92	-0.71	1.29	1.21	1.2
S742	0.25	0.34	-0.08	-0.19	0.62	0.7	0.43	0.37	-0.25	0.23	0.54	0.35	-0.3	0.13	-0.41	0	0.18	0.23	0.53	0.56
I761	0.83	0.11	1.77	1.22	-0.9	1.98	0.52	0	1.02	-1.08	-0.75	1.08	1.78	0.59	0.22	1.29	0.74	-0.71	-0.19	-0.09
Y768	1.83	0.61	1.29	1.52	0.92	1.75	0.25	-0.3	0.85	-0.18	-0.23	0.1	2.75	-1.19	0.4	1.56	0.67	0.1	0.75	0
H769	0.48	0.37	-0.03	0.35	0.49	0.94	0	0.5	0.36	0.4	0.5	-0.09	-0.08	0.42	0.39	0.15	0.11	0.51	0.48	0.55
A770	0	0.62	1.3	1.38	0.86	1.67	1.12	0.55	1.48	0.53	0.66	1.43	0.13	1.15	0.97	0.99	1.07	0.45	0.43	0.71
N771	0.05	0.07	-0.36	-0.09	0.37	0.62	0.03	0.42	0.23	0.44	0.48	0	0.19	0.21	0.33	-0.47	-0.35	0.22	0.44	0.3
Q772	1.58	1.17	0.77	0.87	0.42	1.85	0.33	0.91	0.98	0.46	0.49	0.55	1.96	0	0.7	1.15	0.79	1.03	0.54	0.45
K787	0.85	1.25	-0.63	-0.28	0.79	1.33	0.32	0.99	0	0.5	0.6	0.61	1.76	0.12	0.11	0.8	0.52	0.91	0.67	0.6
M791	0.47	1.06	0.58	0.07	0.43	1.39	0.36	-0.37	0.2	-0.41	0	0.69	1.41	-0.02	0.03	0.66	0.49	0.6	0.38	0.39
T801	0.03	-0.03	0.19	0.33	0.23	0.42	0.24	-0.34	0.35	0.14	0.09	0.13	-0.55	0.35	0.05	-0.02	0	-0.11	0.07	0.28
H805	0.09	0.42	0.12	-0.09	0.4	1.11	0	0.23	0.04	0.3	0.23	0.33	0.49	-0.01	-0.02	0.2	0.4	0.45	0.31	0.31
G823	0.33	0.29	0.52	0.76	0.77	0	0.61	1.2	1.07	0.91	0.68	0.39	1.23	0.78	1.03	0.61	0.94	0.93	1.01	0.79
D843	-0.04	0.13	0	-0.31	0.31	0.74	0.23	0.13	0.44	0.36	0.35	0.28	0.57	0.12	0.34	-0.03	0.02	0.02	0.51	0.42
A846	0	0.06	0.71	0.63	0.49	1.34	0.93	1.06	-0.63	0.9	0.9	0.8	1.62	0.51	-0.42	0.58	0.82	0.12	0.57	0.38
Y864	-0.17	0.47	0.29	0.35	-0.02	0.93	-0.29	-0.16	-0.52	0.25	-0.06	0.26	0.22	-0.26	0.04	0.22	0.22	0.11	0.06	0
T872	-0.08	0.94	0.22	0.29	0.55	1.14	0.38	-0.12	0.46	-0.26	0.63	0.5	-0.01	0.19	0.32	0.42	0	0.42	0.4	0.52

NOTE: The horizontal axis of the table represents the mutated amino acids; the vertical axis represents the mutation sites of NST-M1. The values indicate the change in Gibbs free energy before and after mutation ($\Delta\Delta G$), unit is KJ/mol.

Supplementary Table 4. Analysis of disaccharide composition of different heparins by PAMN-HPLC.

	ΔUA-*p*NP	ΔUA-GlcNAc	ΔUA-GlcNS	ΔUA-GlcNAc6S	ΔUA2S-GlcNAc	ΔUA-GlcNS6S	ΔUA2S-GlcNS	ΔUA2S-GlcNAc6S	ΔUA2S-GlcNS6S
UFH	N.D.	7.39%	0.31%	6.29%	2.60%	14.23%	4.79%	1.22%	63.13%
HS	N.D.	20.11%	0.29%	13.03%	18.98%	9.51%	3.18%	0.44%	34.42%
Enoxaparin	N.D.	11.95%	0.29%	6.85%	2.62%	11.73%	3.36%	0.02%	63.14%
Nadroparin	N.D.	15.62%	3.14%	8.20%	1.64%	8.64%	2.41%	0.07%	60.24%
Dalteparin	N.D.	11.07%	1.97%	7.26%	0.53%	8.86%	2.07%	N.D.	68.20%
Fondaparinux	N.D.	N.D.	N.D.	N.D.	N.D.	N.D.	N.D.	N.D.	100%
Dekaparin	16.06%	N.D.	N.D.	N.D.	N.D.	N.D.	N.D.	N.D.	83.93%
K5EH	N.D.	4.44%	0.24%	2.35%	2.31%	10.07%	3.03%	0.03%	77.50%

NOTE: The horizontal axis of the table represents the composition of disaccharides; the vertical axis represents different heparins. The values indicate the composition ratio of disaccharides analyzed by PAMN-HPLC. N.D. indicates the disaccharide component was not detected (<0.01%).

Supplementary references

1. Wang, Y.-J. *et al.* Imaging of Escherichia coli K5 and glycosaminoglycan precursors via targeted metabolic labeling of capsular polysaccharides in bacteria. *Science Advances* **9**, eade4770 (2023).
2. Sheng, J., Xu, Y., Dulaney, S. B., Huang, X. & Liu, J. Uncovering biphasic catalytic mode of C5-epimerase in heparan sulfate biosynthesis. *Journal of Biological Chemistry* **287**, 20996–21002 (2012).
3. Liu, C. *et al.* Molecular mechanism of substrate specificity for heparan sulfate 2-*O*-sulfotransferase. *Journal of Biological Chemistry* **289**, 13407–13418 (2014).
4. Kitagawa, H., Fujita, M., Ito, N. & Sugahara, K. Molecular cloning and expression of a novel chondroitin 6-*O*-sulfotransferase. *Journal of Biological Chemistry* **275**, 21075–21080 (2000).
5. Edavettal, S. C. *et al.* Crystal structure and mutational analysis of heparan sulfate 3-*O*-sulfotransferase isoform 1. *Journal of Biological Chemistry* **279**, 25789–25797 (2004).
6. Sheng, A. *et al.* Coupling liquid chromatography and tandem mass spectrometry to electrophoresis for in-depth analysis of glycosaminoglycan drugs: heparin and the multicomponent sulodexide. *Anal. Chem.* **93**, 1433–1442 (2021).
