# Supplementary material for: Biosynthetic production of anticoagulant heparin polysaccharides through metabolic and sulfotransferases engineering strategies
Source: Nat Commun. 2024 May 4;15:3755. doi: 10.1038/s41467-024-48193-5 (PMC11069525; doi:10.1038/s41467-024-48193-5)
Supplement: Supplementary file 3 — Description of Additional Supplementary Files [file 41467_2024_48193_MOESM3_ESM.pdf]

Description of Additional Supplementary Files

File Name: Supplementary Data 1
Description: Primer sequences used in this study

File Name: Supplementary Data 2
Description: Screening results of simplified codon and combined mutation library
