# Supplementary material for: Biosynthetic production of anticoagulant heparin polysaccharides through metabolic and sulfotransferases engineering strategies
Source: Nat Commun. 2024 May 4;15:3755. doi: 10.1038/s41467-024-48193-5 (PMC11069525; doi:10.1038/s41467-024-48193-5)
Supplement: Supplementary file 6 — Reporting Summary [file 41467_2024_48193_MOESM6_ESM.pdf]

nature portfolio

Corresponding author(s): Ju-Zheng Sheng

Last updated by author(s): Dec 25, 2023

Reporting Summary

Nature Portfolio wishes to improve the reproducibility of the work that we publish. This form provides structure for consistency and transparency in reporting. For further information on Nature Portfolio policies, see our Editorial Policies and the Editorial Policy Checklist.

Statistics

For all statistical analyses, confirm that the following items are present in the figure legend, table legend, main text, or Methods section.

n/a	Confirmed	
☐	☒	The exact sample size (*n*) for each experimental group/condition, given as a discrete number and unit of measurement
☐	☒	A statement on whether measurements were taken from distinct samples or whether the same sample was measured repeatedly
☐	☒	The statistical test(s) used AND whether they are one- or two-sided<br>*Only common tests should be described solely by name; describe more complex techniques in the Methods section.*
☒	☐	A description of all covariates tested
☒	☐	A description of any assumptions or corrections, such as tests of normality and adjustment for multiple comparisons
☐	☒	A full description of the statistical parameters including central tendency (e.g. means) or other basic estimates (e.g. regression coefficient) AND variation (e.g. standard deviation) or associated estimates of uncertainty (e.g. confidence intervals)
☒	☐	For null hypothesis testing, the test statistic (e.g. *F*, *t*, *r*) with confidence intervals, effect sizes, degrees of freedom and *P* value noted<br>*Give P values as exact values whenever suitable.*
☒	☐	For Bayesian analysis, information on the choice of priors and Markov chain Monte Carlo settings
☒	☐	For hierarchical and complex designs, identification of the appropriate level for tests and full reporting of outcomes
☒	☐	Estimates of effect sizes (e.g. Cohen's *d*, Pearson's *r*), indicating how they were calculated

Our web collection on statistics for biologists contains articles on many of the points above.

Software and code

Policy information about availability of computer code

Data collection	SWISS-MODEL was used for homology modeling. The NCBI Protein Database was used to retrieve protein sequences. Molecular dynamics simulations were conducted. SnapGene was used for computer-aided primer design. The PROSS server was employed for predicting mutations. Rosetta_ddg was used for Virtual saturation mutation calculation.
Data analysis	LabSolutions software was used for analyzing HPLC data. OpenLAB CDS and Thermo Xcalibur software were used for analyzing LCMS data. Schrödinger was utilized for protein structure visualization. Microsoft Excel, ChemDraw 2019, Origin 2021, and Adobe Illustrator 2020 were used for data analysis and graphical visualization.

For manuscripts utilizing custom algorithms or software that are central to the research but not yet described in published literature, software must be made available to editors and reviewers. We strongly encourage code deposition in a community repository (e.g. GitHub). See the Nature Portfolio guidelines for submitting code & software for further information.

Data

Policy information about availability of data

All manuscripts must include a data availability statement. This statement should provide the following information, where applicable:

- Accession codes, unique identifiers, or web links for publicly available datasets
- A description of any restrictions on data availability
- For clinical datasets or third party data, please ensure that the statement adheres to our policy

The author declares that all data supporting the findings of this study are available in the paper and its supplementary information files, or can be obtained from the corresponding author upon reasonable request. The abstract of the report is provided as supplementary information. The datasets generated and analyzed during the current study are available from the corresponding author upon request. The source data is provided as supplementary data.

Research involving human participants, their data, or biological material

Policy information about studies with human participants or human data. See also policy information about sex, gender (identity/presentation), and sexual orientation and race, ethnicity and racism.

Reporting on sex and gender	N/A
Reporting on race, ethnicity, or other socially relevant groupings	N/A
Population characteristics	N/A
Recruitment	N/A
Ethics oversight	N/A

Note that full information on the approval of the study protocol must also be provided in the manuscript.

Field-specific reporting

Please select the one below that is the best fit for your research. If you are not sure, read the appropriate sections before making your selection.

☒ Life sciences ☐ Behavioural & social sciences ☐ Ecological, evolutionary & environmental sciences

For a reference copy of the document with all sections, see nature.com/documents/nr-reporting-summary-flat.pdf

Life sciences study design

All studies must disclose on these points even when the disclosure is negative.

Sample size	Data was analyzed from 3 replicates (sample size) based on the standard sample size used for statistical calculations in biological experiments.
Data exclusions	No date were excluded from analysis.
Replication	Reproducibility was verified using three independent replicates for each data point. The average value was reported in the manuscript, and the standard deviation of the data was calculated and presented as error bars.
Randomization	The selection of transformants from the mutant library was carried out by randomly picking individual, well-isolated transformants on the transformation plate.
Blinding	Not applicable. No data randomization was performed.

Reporting for specific materials, systems and methods

We require information from authors about some types of materials, experimental systems and methods used in many studies. Here, indicate whether each material, system or method listed is relevant to your study. If you are not sure if a list item applies to your research, read the appropriate section before selecting a response.

Materials & experimental systems

n/a	Involved in the study
☒	☐ Antibodies
☒	☐ Eukaryotic cell lines
☒	☐ Palaeontology and archaeology
☒	☐ Animals and other organisms
☒	☐ Clinical data
☒	☐ Dual use research of concern
☒	☐ Plants

Methods

n/a	Involved in the study
☒	☐ ChIP-seq
☒	☐ Flow cytometry
☒	☐ MRI-based neuroimaging

Plants

Seed stocks	N/A
Novel plant genotypes	N/A
Authentication	N/A
